# Regulatory Barriers to Innovative Plant Breeding in Canada

**DOI:** 10.3389/fgeed.2020.591592

**Published:** 2020-10-20

**Authors:** Stuart J. Smyth, Savannah Gleim, Simona Lubieniechi

**Affiliations:** Department of Agricultural and Resource Economics, University of Saskatchewan, Saskatoon, SK, Canada

**Keywords:** gene editing, investment, plants with novel traits, regulatory uncertainty, risk

## Abstract

The regulation of plant breeding is gaining increasing scrutiny, particularly as it pertains to the regulation of gene editing and other new breeding technologies. Genome editing is used worldwide in both public and private plant breeding laboratories and there is considerable uncertainty about the ability of regulatory agencies to match the rapid scientific pace being set. This research focuses on Canada, where advances in plant breeding technology are constrained by the boundaries of the regulatory system established in the early 1990's. This research presents the results of a survey of 93 public and private plant breeders and their views on the existing Canadian regulatory framework regarding conventional breeding and genome editing techniques for plants with novel traits (PNTs). The results contribute to the ongoing debate regarding how, or whether, to regulate products of genome-edited plant breeding, beyond the existing agronomic and safety requirements. Plant breeders identify the level of Canadian crop research competitiveness and quantify the impacts of novelty within Canada's regulatory system for PNTs. One significant finding is that PNT regulations in Canada have created an innovation barrier in terms of applying genome editing technologies to the development of new varieties, particularly in public sector research.

## Introduction

Regulatory uncertainty is one of, if not the, leading determinant for global research and development (R&D) investment. The uncertainty caused by delays in obtaining regulatory approval for new crop varieties reduces the return on investment to such a degree that firms will not make technology development investments (Smyth et al., 2014). Additional reasons for regulatory uncertainty can include the lack of clarity in some countries regarding the requisite data required for approval, the need to gather data on socio-economic considerations and potential trade concerns once the variety is approved. The regulatory uncertainty that exists within the European Union (EU) regarding the approval of genetically modified (GM) crops has resulted in multinational companies relocating much of their agricultural R&D activities to jurisdictions more conducive to the commercialization of these crop technologies (Smyth, 2019). It has never been more important for countries to have efficient regulatory systems, given the importance of regulatory competitiveness and attracting global R&D investment.

Canada has been a leading country in terms of the development and adoption of GM crops, having made 123 regulatory approval decisions between 1995 and 2019 (Canadian Food Inspection Agency, 2020). Canada's science-based regulatory system was developed with input from scientists working as regulators, academics and in industry. Regulatory approval decisions are based on the final product, not the process used to create the product. The Canadian regulatory system developed in the early 1990's regulates plants with novel traits (PNTs), comparing them to conventional varieties. PNT varieties are approved if a risk assessment concludes the risk of the PNT variety is substantially equivalent to conventional crop varieties.

Food security was identified as a core objective by the G7 Ministers of Agriculture in 2016, when they committed to improving sustainable agricultural production, productivity and food supply through a combination of research and governance innovations. Without a doubt, there is a need for more agricultural R&D investment, particularly private sector investment. The most significant challenge is that <1% of agri-food innovations succeed (Graff et al., 2009) and the costs and time to get new products commercialized are increasing and becoming more uncertain in Canada and abroad (Phillips McDougall, 2011). Canada has long been recognized as a global leader in terms of R&D investment and the levels of high quality science being conducted. However, Canada consistently suffers from a commercialization lag, whereby outputs from Canada's innovation system are deemed to be low, when compared to innovation system inputs. The gap between innovation capability and commercialization is illustrated by the Global Innovation Index (2018) which ranked Canada 61st in terms of innovation efficiency, comparing innovation outputs to innovation investments. Canada is positioned as the second-lowest of the top 20 economies in terms of innovation efficiency. The cost of regulation in Canada is reflected through the Global Competitiveness Index (World Economic Forum, 2019) ranking of Canada in the 53rd position in terms of the burden of government regulation.

The objective of this article is to assess whether Canada's PNT regulatory framework is viewed as a barrier to innovation by Canadian public and private plant breeders. The survey was intentionally designed to be broad, including any plant breeding technology capable of creating a PNT variety. A survey of nearly 100 plant breeders carried out in 2018, found that plant breeders are strongly of the view that the present regulatory system is a barrier to innovation.

## Background

Barriers to innovation can be both technological and regulatory. Technological barriers are commonly the easier of the two to resolve, as additional investments in innovation and the recruitment of top scientists are capable of advancing new solutions and adapting existing ones. Plant breeding is one area of innovation that has significantly benefited from technological advances as it has moved from random, uncontrolled radiation and chemical mutagenesis for creating useful genetic variation, to genetic modification, to the use of gene editing where controlled and targeted gene mutations are made (Zhang et al., 2017; Wolt and Wolf, 2018; Eriksson, 2019; Eriksson et al., 2019). The technology advances from the improvement in crop breeding have contributed to the reduction in the time of new variety development from 7 to 25 years, down to potentially as few as 2–3 years (Friedrichs et al., 2019). Thus, based on the potential offered by gene editing technologies, technology barriers can be viewed as a minimal constraint on current plant breeding.

Regulatory barriers, on the other hand, are more nebulous and challenging to navigate. Research by Lassoued et al. (2019) estimated that if gene-edited crops are regulated as equivalent to conventional crops, the cost and time of variety development and regulatory approval would be US$10.5 million and 5 years, compared to US$24.5 million and 14 years if developed and regulated as equivalent to genetically modified (GM) crop varieties. Regulatory barriers carry significant costs, both fiscal and time to market. Smyth et al. (2014) identify that regulatory barriers of 6 years reduce the return on investment to zero for private sector investors, while 2-year delays reduce the public sector returns to zero.

Regulatory competitiveness has become a key leading investment indicator for multinational corporations. In 2012, BASF announced it was relocating its agricultural biotechnology research division from Europe, due to regulatory barriers (BASF, 2012). This relocation to North and South America involved 140 staff. The principal reason for this investment strategy change was the 13 years required for BASF to receive regulatory approval for its genetically modified Amflora potato, within the EU. The regulatory delay was so costly that BASF never commercialized the variety, stranding all the capital that had been invested in the development of the potato variety. Further compounding the European Union's decline in regulatory competitiveness was the 2018 decision by the Court of Justice of the EU (CJEU), that crop varieties developed by means of new techniques and/or methods of mutagenesis would be required to be regulated using the EU's regulations for GM crops (Court of Justice of the European Union, 2018).

The impact on EU R&D investment into agricultural innovation was immediate. Within days, BASF announced it would end all investment in gene editing with the EU, a decision that was also announced by Bayer (Reuters, 2018). Having two European-based agricultural multinational firms announce they would no longer invest in agricultural gene editing R&D in their home jurisdictions, further confirms how the EU's regulatory system has become a massive barrier to innovation. Within the months following the CJEU's ruling, the decline in R&D investment began to extend to small and medium-sized plant breeding firms (Sikkema, 2018). The combination of investment reductions from large multinational and small and medium plant breeding firms holds the potential to have a devastating impact on the future of plant breeding in Europe. This is on top of previous investment declines resulting in the percentage of global agricultural R&D investments in the EU, dropping from over 30% in 1995 to <10% in 2015 (Little, 2015).

At the other end of the spectrum, Argentina has a progressive regulatory system concerning gene-edited crop varieties. Research by Whelan et al. (2020) confirms an increase in R&D and variety commercialization by local companies and public research institutions when compared with the development of GM crop varieties. Not only has there been an expansion in the number of stakeholders engaged in gene editing plant research, but the research has additionally expanded to animals and microorganisms. The broadening of gene editing research in Argentina, where the regulatory system facilitates innovation, runs counter to the experience within the EU where companies are exiting the crop research sector, due to the burden of regulation.

Progressive regulatory approaches to innovative technologies provide preferred R&D investment climates, which is further quantified by recent evidence from Canada. Gleim et al. (2020) reporting on the results of a 2018 survey of 93 Canadian plant breeders, found that one-third of public and private plant breeders were using gene editing technologies as part of their current research programs, with two-thirds expecting to use these technologies by 2021. One hundred percent of the public breeders responded they anticipated an easier path to commercialization from utilizing CRISPR, with 88% of private breeders similarly reporting.

Reducing the regulatory burden of innovation is crucial to ensuring future R&D investments in all industries. As the technology of GM crops began to shift from laboratory to field in the late 1980's, Canada developed a regulatory framework for assessing the risks of plants, known as plants with novel traits. PNT status can apply to all crop varieties, regardless of the process used to develop them, as well as traits that did not exist prior to the establishment of PNT regulations. The challenge that has resulted from the establishment of PNT status is that many public variety developers will not develop PNT varieties, as this would require separate development capacities. Separate development processes are essential to ensure no cross-pollination occurs between a PNT variety and a non-PNT variety. The cost to maintain two distinct processes for variety development is beyond the ability of most public variety developers. The PNT distinction can be viewed as a regulatory barrier for the public sector in terms of variety development.

## Methodology and Demographics

A survey commissioned by CropLife Canada, whereby Canadian plant breeders were surveyed to gain their views on the process of developing and regulating crop varieties within Canada. To carry out the research, the survey and analysis, the funds from CropLife Canada were matched by the Plant Phenotyping and Imaging Research Center (P^2^IRC) project, a Canadian First Research Excellence Fund grant held by the Global Institute for Food Security at the University of Saskatchewan. In total 54 questions were asked of respondent through the online platform, Survey Monkey.

Over the course of April and May 2018, 430 potential respondents were emailed extending an opportunity to participate. These respondents consisted of professionals from the Canadian plant breeding sector, which include both private and public breeders, consultants or staff of technology developer companies, public trait developers and researchers. The respondent list was populated through the assistance of CropLife Canada, along with our connections, partners in the P^2^IRC project and general webpage searches of plant breeders and regulatory affairs directories to generate names and an email list. This type of sampling is known as purposive sampling, it is a non-random or non-probability form of sampling in which the results cannot be generalized to the entire population, as sampling restricts the range of the inference. As a result of non-probability sampling, it is not possible to calculate confidence intervals and margins of error. Therefore, our results will only use descriptive statistics and percentages, as a result of our non-random small sample size. The validity of our conclusions cannot be extended to the entire population, but are representative of the Canadian plant breeding sector.

**Figure 1:**
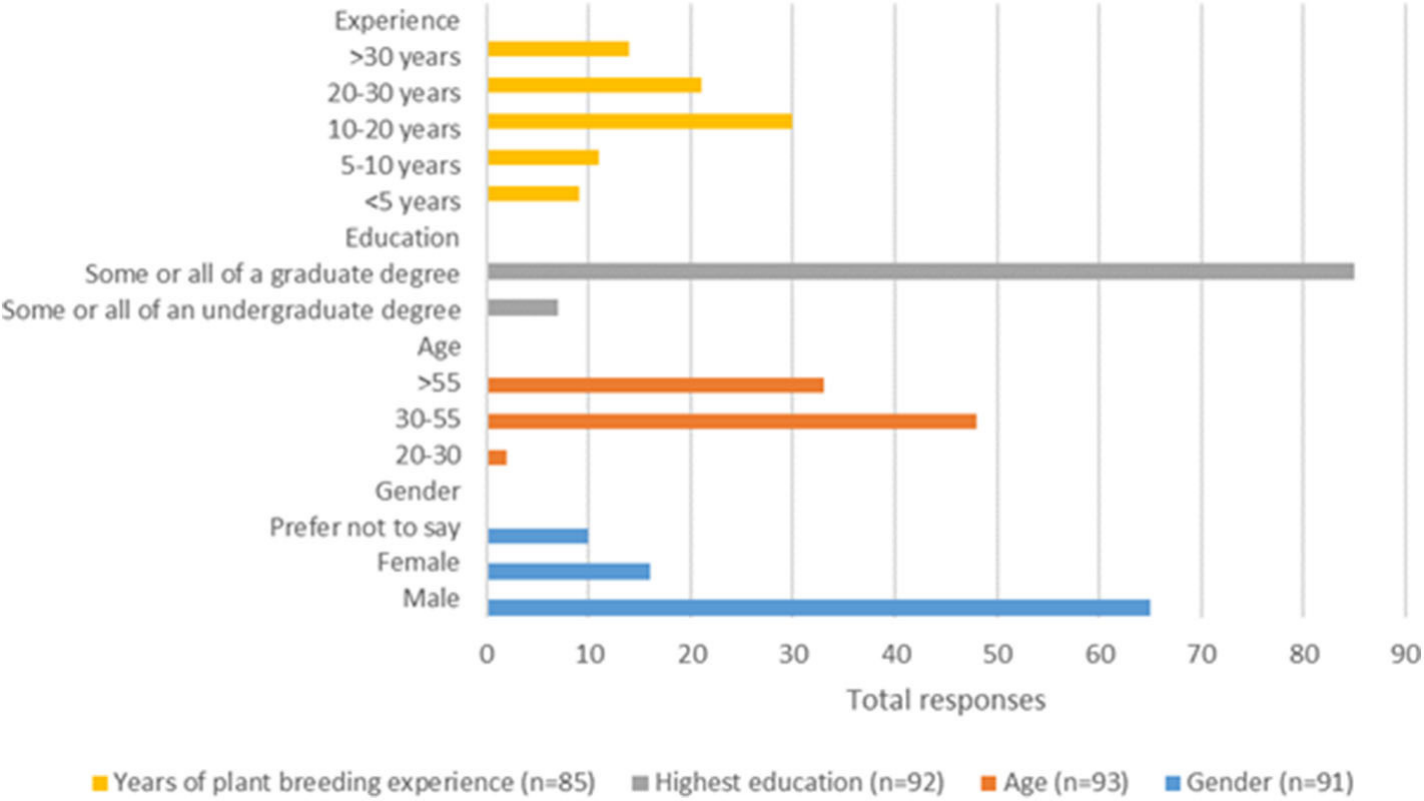
Survey response demographics.

Our online survey was emailed out to 430 individuals, of which 114 submitted responses. For this article, only 93 survey responses were considered, as 21 were incomplete or unassignable, resulting in a 22% survey response rate. Respondents of our survey were predominantly male, accounting for 71%, with 18% female (Figure 1). Over half of the respondents were between the ages of 30–55 years old and 37% were over 55. Given the nature of the discipline being surveyed, it was not unexpected that 92% identified as having completed some, or all, of a graduate degree. Also, having used a list generated by CropLife Canada, and our ability to search out individuals with a webpresence, very few of the respondents were new to plant breeding, with only 11% having <5 years and 13% with 5–10 years accumulated experience. Most respondents had significant plant breeding involvement, with 35% having 10 to 20 years of experience, 25% between 20 to 30 years, and 17% had over 30 years. Of our sample, 42% identified as being a member of the private sector, and the remaining 58% identified as plant breeders of the public sector. The survey sample is characterized as a mature and highly educated population, mostly male, with substantial experience in plant breeding in the public sector.

For the majority of questions, respondents were provided with a five-point Likert scale with choices from “extremely familiar,” “moderately familiar,” “somewhat familiar,” “slightly familiar” and “not familiar at all.” Alterations on this included the same five-point Likert scale with agree instead of familiar, “strongly agree,” “somewhat agree,” “neutral,” “somewhat disagree” and “strongly disagee.” On some questions the option of “don't know” was included.

## Results and Discussion

**Figure 2:**
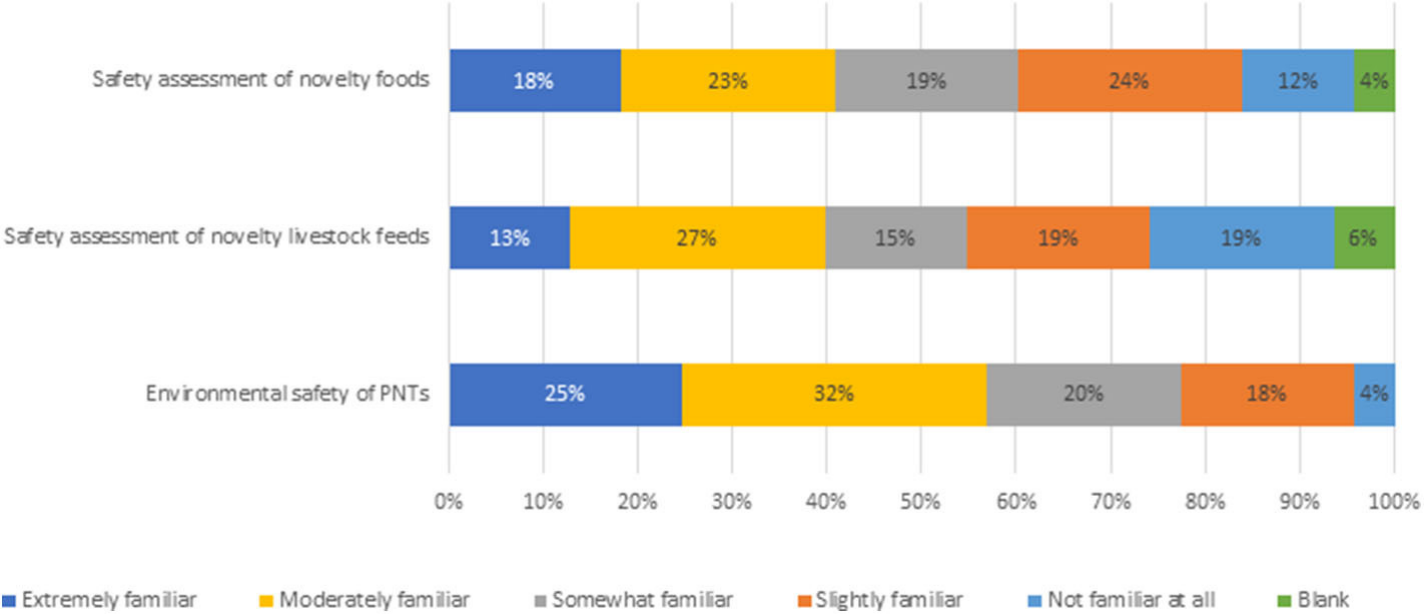
Familiarity with regulations and risk assessment processes in Canada for PNTs (*n* = 93).

To assess whether plant breeders view the PNT regulation framework as a barrier to innovation, three variables needed to first be quantified to ensure those responding were well-informed about the regulations in question. The three variables were familiarity with existing regulations, the cost and timeliness of regulatory approvals and whether existing regulations encourage innovation. First, respondents were asked how familiar they were with the regulations and risk assessment processes for novel products in Canada for the following categories: safety assessment of novel foods, novel livestock feeds and environmental safety of PNTs (Figure 2). Responses varied slightly across the PNT categories, with the greatest familiarity with PNTs' environmental safety assessment, followed by novel foods and novel feeds safety assessment. The response that 19% of plant breeders are not at all familiar with feed regulations is a surprise as in Canada, as all PNT varieties have to be approved for food and feed use. Given that all PNT variety approvals include novel food and feed assessments, the article presents all results based on PNT responses. The same data is used for both assessments, so it is possible that this may explain some of the lack of familiarity. There is no clear indication as to why awareness of feed regulatory requirements are lower than food and environmental safety, however on average, 84% of respondents indicated some level of familiarity, providing confidence in breeder views on perceived regulatory barriers.

**Figure 3:**
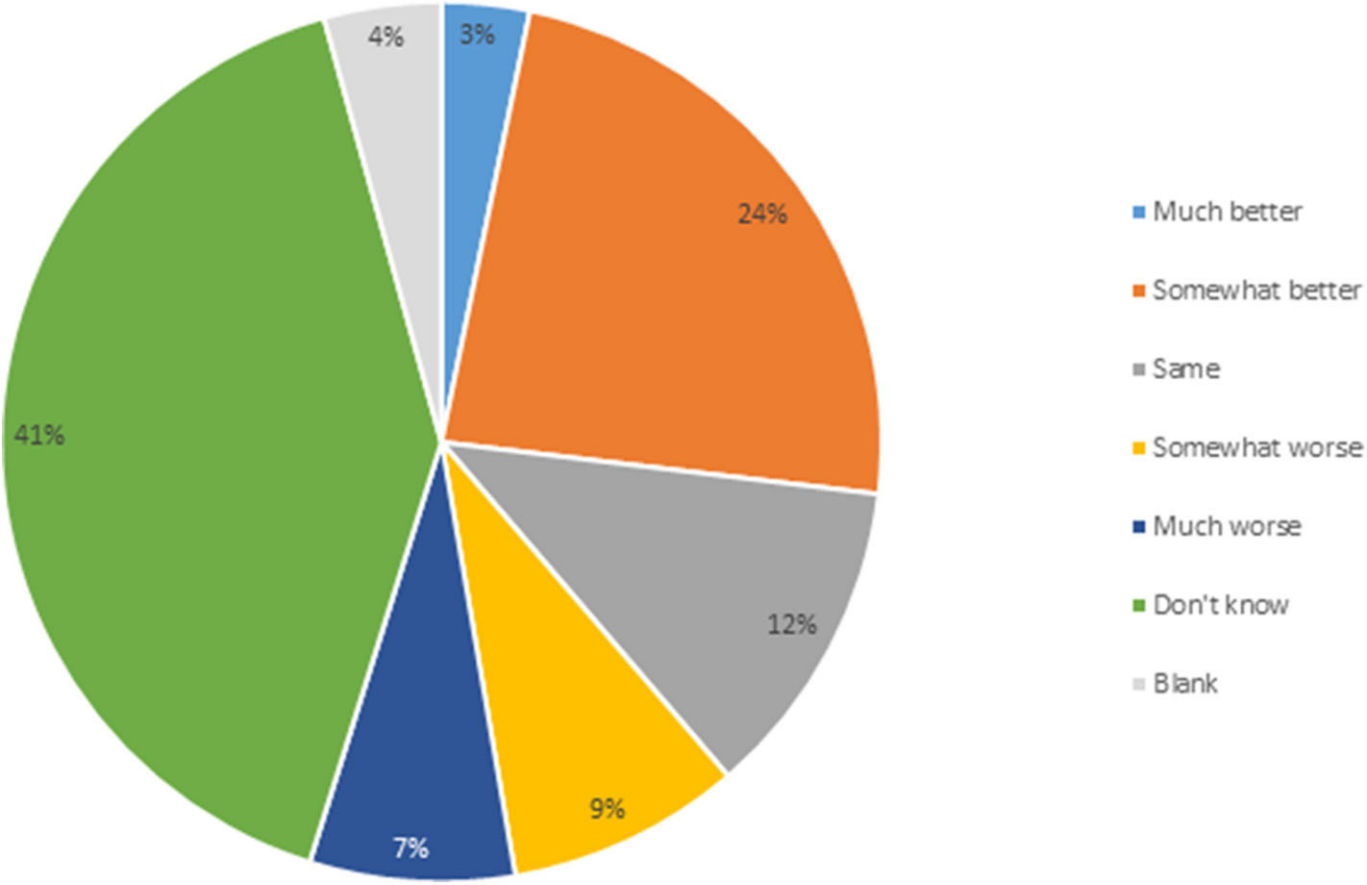
Comparing regulatory costs and timelines for transgenic plant authorization in Canada vs. other jurisdictions (*n* = 93).

Second, respondents were asked how they would rank the regulatory costs and timeliness for the authorization of rDNA plants in Canada compared to other jurisdictions. Twenty-seven percent indicated that Canada is either much or somewhat better than other jurisdictions, with 16% believing Canada is much or somewhat worse (Figure 3). Twelve percent consider regulatory time and cost to be equivalent as in other jurisdictions. While 41% indicated they did not know, this level of response may partially be explained by breeders that breed varieties only for the Canadian market, resulting in them having no foreign market experience to compare with. Commercialization on GM PNTs requires commodity import markets to approve the GM variety for import, adding to the time and cost of variety approval. There has only been one GM variety developed by a public institution and approved in Canada, GM flax in 1998. Some mutagenic varieties regulated as PNTs have resulted in the Canadian commodity industries engaging in dialogue with import markets to reassure them that the PNT status does not mean the variety is a GM variety. Results of the second variable assessment indicate that the majority of respondents are capable of assessing the time and cost of the Canadian regulatory process with that of other jurisdictions.

**Figure 4:**
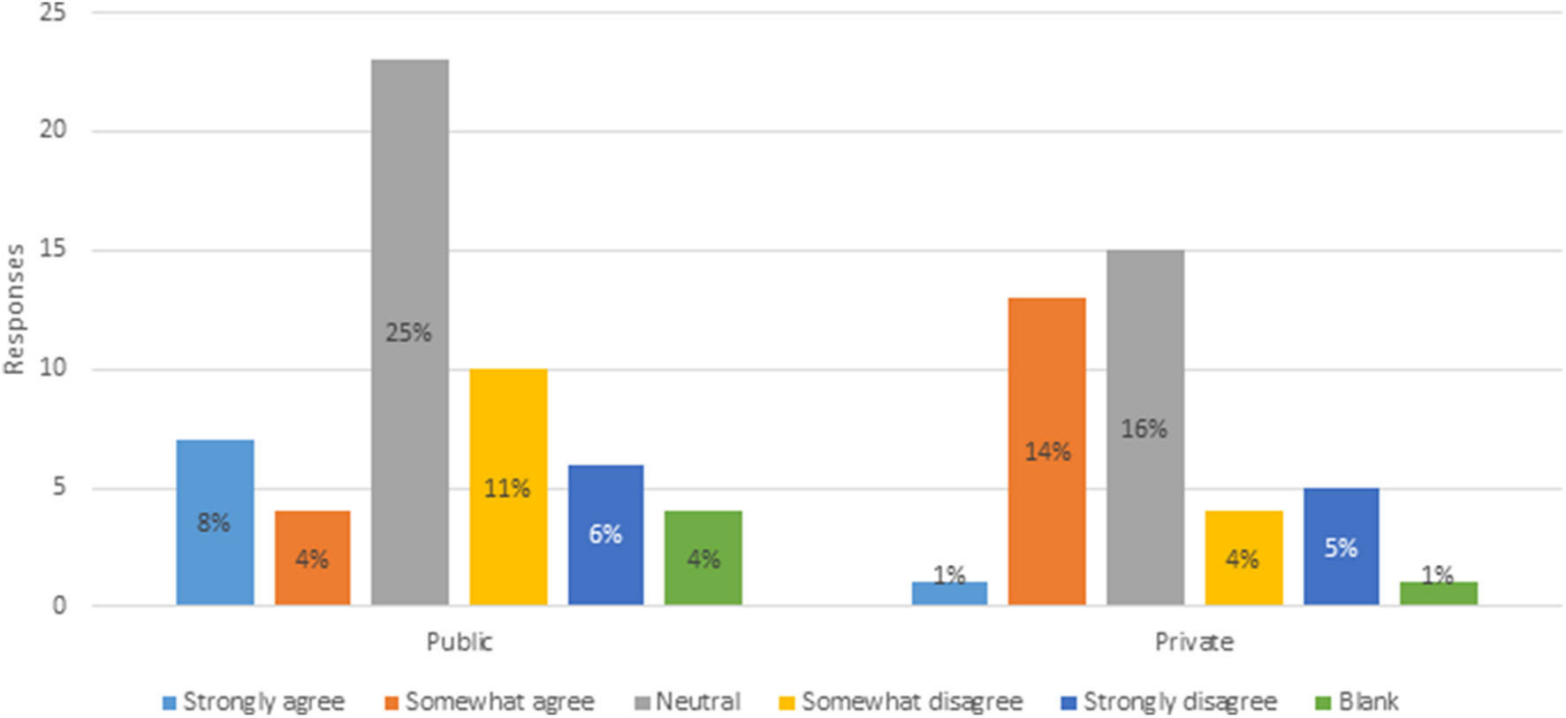
Extend of agreeance that Canada's novelty approach encourages investment and innovation I plant breeding (*n* = 93).

To quantify the third variable, respondents were asked to what extent they agreed or disagreed that Canada's novelty approach encourages investment and innovation in plant breeding. Figure 4 indicates that 12% of public and 15% private breeders somewhat or strongly agree, compared to 17% of public and 9% of private breeders that somewhat or strongly disagree. Private breeders are more inclined to agree that Canada's novelty approach encourages investment and innovation, while public breeders are more inclined to disagree. Forty-one percent of respondents were neutral. Assessment of these variables confirms that respondents are aware of, and knowledgeable about, Canada's PNT regulatory framework, providing confidence in the remainder of the data responses.

In Canada, the Canadian Food Inspection Agency (CFIA) and Health Canada are the regulators of novel crops, therefore respondents were asked to what extent they believed there are clear guidelines provided by the CFIA and Health Canada, which allows them to independently determine if a new variety they wish to develop, or they are developing, will be regulated as a PNT. A lack of clarity reduces a breeder's ability to move their novel crops through the regulatory framework. Nearly half of the respondents (48%) indicated the guidelines are somewhat or very clear, compared to 26% that indicated guidelines were somewhat or very unclear. A further 9% responded that the guidelines are neither clear nor unclear, while 13% did not know.

To better assess if plant breeders viewed PNT regulations as a barrier to their research and innovation, they were asked to express their perspective regarding several statements concerning their research. First, they were asked to respond to the statement: “To accelerate my research project, I have conducted or considered conducting field research outside of Canada to avoid regulation under the confined research field trial for PNTs” (Table 1). The majority of respondents (63%) disagreed with this statement, with 27% indicating they have accelerated their research outside of Canada. Of the public breeders responding to this statement, 40 out of 54 indicated they have not undertaken field research outside of Canada. The high proportion of public breeders indicating they do not engage in accelerating their breeding programs outside of Canada may also be due to the lack of fiscal resources to undertake collaborations or the lack of available collaborators.

**Table 1:** Perspectives regarding Canadian regulations (*N* = 93).

	**Public sector**				**Private sector**			
	**Agree**	**Disagree**	**Don't know**	**No response**	**Agree**	**Disagree**	**Don't know**	**No response**
To accelerate my research project, I have conducted or considered conducting field research outside of Canada to avoid regulation under the confined research field trial for PNTs.	8%	44%	N/A	6%	19%	19%	N/A	3%
Science-based development, evaluation and commercialization of Canadian innovations has provided a competitive advantage for Canadian products in the world market.	22%	9%	25%	3%	10%	6%	18%	8%
I have transfered germplasm from a Canadian R&D project to collaborators outside of Canada in order to accelerate commercialization.	3%	45%	0%	10%	6%	18%	0%	17%
The regulatory requirements need to be updated as we learn more about the safe use of technology.	46%	0%	6%	5%	31%	2%	6%	2%

In the second statement respondents were asked whether: “science-based development, evaluation and commercialization of Canadian innovations have provided a competitive advantage for Canadian products in the world market.” Respondents were twice as likely to agree, than disagree, as 32% agreed, while 15% disagreed. In total, 43% of respondents did not know whether or not there was an advantage.

When asked whether they had transferred germplasm to research partners or collaborators outside of Canada to accelerate commercialization, very few respondents indicated they had done so, with only 9% agreeing to having done this. Responses indicate that the practice is more common with private sector breeders than public.

Plant breeders had strong opinions regarding the statement: “The regulatory requirements need to be updated as we learn more about the safe use of technology.” A substantial majority (77%) of the respondents agreed there was a need to update PNT regulations to better reflect current technologies. Only two individuals disagreed with the statement.

To determine if PNT regulations are having an impact on present variety development research, respondents were asked for their views on three statements. The first statement: “At least one of my research proposals has been turned down due to uncertainty about the product's potential novelty during the development and evaluation phase” (Table 2), shows slightly more respondents disagreed (34%), than agreed (22%). When asked whether they had had research proposals rejected “due to uncertainty about the regulatory costs in commercialization and marketing,” 29% felt this was true for one of their PNT research proposals, compared to 30% who disagreed. Amongst the two sectors, the public sector was nearly split with 17 individuals agreeing, 20 disagreeing and the remaining 17 did not know or did not respond. Of the 39 private sector responses, 22 of these private sector individuals did not respond or know at all. While Canada's regulatory system does not involve non-science aspects, such as consumer acceptance, respondents were probed whether they thought research was rejected “due to uncertainty about the public acceptance of a GM product.” Slightly more disagreed (30%), than agreed (25%).

**Table 2:** PNT regulation impacts on research proposals (*N* = 93).

**At least one of my research proposals has been turned down…**	**Public sector**				**Private sector**			
	**Agree**	**Disagree**	**Don't know**	**No response**	**Agree**	**Disagree**	**Don't know**	**No response**
…due to uncertainty about the product's potential novelty during the development and evaluation phase.	14%	24%	12%	9%	8%	10%	15%	10%
…due to uncertainty about the regulatory costs in commercialization and marketing.	18%	22%	12%	6%	11%	8%	14%	10%
…due to uncertainty about the public acceptance of a GM product.	19%	19%	11%	9%	6%	11%	14%	11%

As identified above, uncertainty is a key determinant for investment and when the level of uncertainty becomes too great, investments will not be made. Approximately 30% of plant breeders in Canada are expressing that the level of regulatory uncertainty in Canada is negatively affecting innovative R&D proposals. Additionally, it confirms the low ranking of Canada by the Global Competitiveness Index for the burden of government regulation. One-quarter of proposed plant breeding research is being rejected due to the perceived regulatory burden and the uncertainty of the regulatory approval cost, which is of great concern for innovation in Canada.

The objective of this article is to assess plant breeders views on Canada's regulation of novelty varieties, and whether they perceive them to be barriers to innovation. Given this, we asked whether breeders altered their research or carried out further research as a result of regulatory barriers. When asked whether they “decided not to undertake a research proposal or develop an innovation because [they] self-determined the innovation would be considered novel,” 48% disagreed (Table 3). One-third of the respondents agreed that they did not pursue research once they determined that it would be regulated as novel, of which 22% were from the public and 12% from the private industry. Fewer agreed to having altered their “breeding objective to avoid having a product reviewed as novel in Canada,” with 19% agreeing to the statement. While the majority of the respondents disagreed with the statements to not pursue research or altered their breeding objective once they determined the project to be deemed novel, however, 34% and 19% did agree with this statement. One-third and one-fifth of respondents identifying that changing the course of their research due to its “novelty,” suggests that to some extent, the application of novel is a barrier to innovative research. Again, more than one-quarter of the plant breeding research that has been initiated has been internally halted due to novelty determination. While it was not possible to quantify the fiscal costs of these stranded research projects, based on the amount of annual plant science funding in Canada, a conservative estimate would place this cost in the millions of dollars.

**Table 3:** Respondents decisions to alter or extend research due to Canadian regulation barriers (*N* = 93).

	**Public sector**			**Private sector**		
	**Agree**	**Disagree**	**No response**	**Agree**	**Disagree**	**No response**
I decided not to undertake a research proposal or develop an innovation because I self-determined the innovation would be considered novel.	22%	31%	5%	12%	17%	13%
I altered my/our breeding objective to avoid having a product reviewed as novel in Canada.	15%	32%	11%	4%	20%	17%
I had a product in a pathway to commercialization in Canada, but I did not proceed as I found out the product would be considered novel.	3%	38%	17%	3%	22%	17%
I conducted extra research to provide evidence to Canadian regulators that our innovation should not be considered novel.	8%	31%	19%	13%	15%	14%
I experienced delays introducing a new variety in Canada compared to other markets due to the novelty trigger.	9%	27%	23%	9%	16%	17%

One indication of positive news is the responses to the third statement about whether commercialization was halted, when novelty determination was made, with only 6% agreeing and the majority of 60% disagreeing. This may indicate that as the closer the plant variety gets toward commercialization, the level of uncertainty declines. As lines of potential new crop varieties proceed though the innovation pipeline, from greenhouses to field trials, novelty determination is being made at, or before, field trials commence.

When asked about whether or not plant breeders “conducted extra research to provide evidence to Canadian regulators that [their] innovation should not be considered novel,” nearly half of respondents indicated their organization or firm had not conducted further research. Only 21% of respondents admitted to carrying out further research, which is an additional cost, both in terms of fiscal resources invested in undertaking the additional research, as well as the time required.

The last statement that respondents were asked to express their expert opinion on was: “I experienced delays introducing a new variety in Canada compared to other markets due to the novelty trigger.” Similar to the previous responses, less than one-fifth agreed to experiencing a barrier to the market due to a delay. Of those who agreed, fifteen respondents estimated the time of delay: six respondents indicated a year or less; four respondents said that the time delay was between 1 and 3 years; and four respondents indicated that it was more than 5 years. When asked about the fiscal costs of the delays, only 11 respondents estimated the costs of delay. While they had troubles estimating an exact cost, from a few hundred dollars to several millions, several individuals suggested the greatest cost was time. Given that plant breeding research is a complex process, calculating a cost is difficult to do and would vary based on the crop, time, public or private firm, and the size of the breeding program. However, of those who responded, none of them responded that this delay was cost-less. Based on this survey, plant breeders indicate they have experienced delays, but struggle to quantify this in either fiscal or time delay costs.

What is most evident of these three responses is both the high percentage disagreeing with the statements, ranging in the 43–60%, while 18–40% chose not to respond. As previously mentioned, disagreeance could suggest breeders have not experienced such barriers, or perhaps it suggests their acceptance of the existing regulations. However, the high percentage of not responding leads us to question whether they are indifferent, or not willing to state how regulations have influenced their research.

## Conclusions

This survey of Canadian public and private plant breeders reveals there is cause for some concern regarding PNT regulations acting as a barrier to innovation. The strongest indication of this is evidenced by the 77% of respondents indicating that Canada's PNT regulatory framework needs to be updated to reflect current levels of knowledge and the advancements in plant breeding technologies. Supporting the importance of reviewing PNT regulatory requirements are the results that found:

- 22% experienced research proposals being turned down due to PNT uncertainty;
- 34% of breeders have ended research when self-determination indicated PNT status;
- 19% have altered research to ensure the variety was not deemed to be a PNT;
- 18% experienced a delay once PNT status was applied; and
- 26% disagreed that PNT regulations encourage investment.

More than one-quarter of plant breeders are clearly expressing they view PNT regulations to be a barrier to investment. Ultimately, what public breeders are doing is undertaking breeding programs that have as little novelty as is possible, thus ensuring they are not regulated as PNTs. This raises questions of what adoption rates these minimally novel varieties might have once approved and commercialized. Will producers be willing to adopt new varieties that are only marginally better than existing ones? While there is strong support for Canada's science-based regulatory framework, with 32% expressing the belief this provides a competitive advantage, concerns arise when 27% indicate they conduct field trials outside of Canada to avoid the confined field trial requirements that pertain to PNT varieties.

When the results are divided into public and private sector responses, in most questions, it is public breeders agreeing that existing PNT regulations are posing a barrier. As mentioned above, most public sector plant breeders are unable to develop PNT varieties due to the inability to have parallel development infrastructure, due to space, time and cost. A significant percentage of respondents chose not to respond to some questions, which may be due to the lack of public sector experience in developing PNT varieties.

With agricultural R&D investments fleeing Europe, Canada's strong history of R&D provides an attractive market. However, Canada lags in private sector investment and a review of the PNT regulatory framework may be essential to attracting and increasing the private sector R&D investment in Canada.

## Data Availability Statement

The raw data supporting the conclusions of this article will be made available by the authors, without undue reservation.

## Author Contributions

All authors listed have made a substantial, direct and intellectual contribution to the work, and approved it for publication.

## Conflict of Interest

The authors declare that the research was conducted in the absence of any commercial or financial relationships that could be construed as a potential conflict of interest.
